# The General Structure of the Teeth

**Published:** 1887-04

**Authors:** Carl Heitzmann

**Affiliations:** New York.


AMERICAN JOURNAL

—OF—

DENTAL SCIENCE.

VOL. XX. THIRD SERIES—APRIL, 1887. NO. 12.

ARTICLE I.

### THE GENERAL STRUCTURE OF THE TEETH.

BY DR. CARL HEITZMANN, NEW YORK.

Mr. President and Gentlemen, on March 2 a terrible disaster happened to me and to my family. A beloved daughter of mine, 15 years of age, died of pneumonia. Several weeks of suffering attending and following that calamity broke me down to such an extent that I was quite miserable, both bodily and mentally. Some weeks after this ordeal I went to my dentist, Dr. Bodecker, who had long been familiar with the condition of my teeth, and he said it was perfectly surprising how my teeth had softened down in that short time. I suppose every dentist knows that bodily suffering from an acute disease will soften the teeth in a comparatively short time, in the space of but a few weeks; indeed a very prominent dentist of New York, when I inquired about these matters, told me that he is able to tell by working on the teeth of a lady whether she is suffering from womb disease or not. What does that prove? First, that there is a circulation of matter, speaking in a general way, within the structures of the teeth that is very much more lively than was supposed up to our time. It next proves that such circulation of pabulum or of matter being present in the teeth, they must be alive through and through. Now is the season of raspberries and blackberries, and it very often happens that a small seed is caught in a pit of the crown of a tooth, and every one of us has experienced the unpleasant, nay painful, sensation produced when the surfaces of the teeth touch such a seed as they glide over each other, and we know that a small hair being caught between the gliding surfaces of the teeth can be recognized as such. There is a degree of sensibility in the teeth that is truly surprising. What does that prove? Where there is sensibility and sensation there is life. Therefore our teeth are composed of live tissues. These are considerations which necessarily urge us to investigate the question: Where is this life located? Which is the substance that is the bearer of life? It cannot be denied that large quantities of both organic and inorganic matter are stored up in the teeth; but there must be something else, which we may call living matter proper, and though we cannot watch and study the changes in its form directly by comparison with other tissues of the body, we must come to the conclusion that we have to deal with living matter. During the last six or seven years this question has been very actively investigated in my laboratory, and I am now giving you a brief outline of the researches made there, mainly by Dr. Bodecker.

As long as the old method was followed, of drying the teeth and making thin slabs for microscopic examination, mounted in Canada balsam, not the least idea could be obtained as to the living matter within the teeth. This, being distributed through the more solid substances of the tooth, it dried out. You could not determine where the living matter was located, and as to its presence or absence we had not the least idea indeed. It is since the method of examination has been changed that we have reached the degree of perfection which enables us to see the living matter, partly without and partly with the application of certain re-agents. This new method is to soften the teeth by extracting the lime salts with a dilute chromic acid solution, which at the same time preserves the soft tissues or living matter. Another method is to grind a fresh tooth, keeping it moist and preserving the living matter either with chromic or osmic acid. Then if we place a thin section under the microscope we may trace the living matter in it.

Examining the general structure of the teeth we find that there are four varieties of tissue, the main portion of the teeth being composed of dentine, its centre being hollowed out and occupied by a myxomatous tissue, supplied with blood-vessels and nerves, the roots being covered with a structure which we call cementum, and the crown with another structure called enamel. We perceive, by looking at the dentine with a low power, that it is traversed by canaliculi, which were thought to be carriers of lime salts until the discovery was made that they are not only hollow but contain in their centre a solid thread, the dentinal fibre. This latter, curiously enough, was first seen by a draftsman employed by the celebrated Richard Owen, who represented it by a dot in the centre of a transverse section of the dentinal canaliculi, whereas Richard Owen himself does not make any allusion to it in the text of his Odontography. It was the elder Tomes who first described these fine threads and he at the same time expressed the opinion that they might be nervous in nature. Though not positive in this assertion, he suggests the possibility of these fibres being nerves, which may account for the very marked sensibility of the dentine. You know that these fibres in the centre of the dentinal canaliculus are not perfectly round, but angular, and are evidently surrounded by some liquid. If we use a re-agent for bringing out the living matter in the different tissues, namely, chloride of gold, we very soon come to the conclusion that this cross section of the dentinal fibrilla is by no means smooth on its circumference, but that it sends out off-shoots toward the wall of the dentinal canaliculus. Along the periphery of the latter we notice very small perforations, traceable into the basis-substance which surrounds the dentinal canaliculus, and entering into a light, delicate network therein. The basis-substance is far more dense at the periphery of the dentinal canaliculi than at some distance between the canaliculi, a fact first brought to our notice by E. Newman. We satisfy ourselves regarding the fact that the fine conical off-shoots of the dentinal fibre are directed toward the perforations in the wall of the canaliculus, and if we have been lucky in applying the chloride of gold solution—Dr. Williams was so fortunate—we can trace the off-shoots into the dentine and see a delicate reticulum throughout the basis-substance of the dentine. Thus we come to the conclusion that the whole basis-substance is pervaded by an extremely delicate reticulum which exhibits all the properties and characteristics of the dentinal fibres and of living matter. Dr. Bodecker proved the presence of this reticulum without directly proving its being living matter. His proof was indirect, inasmuch as we see in every inflammatory process of the dentine that it breaks down to its embryonal condition, splitting up into the medullary elements from which it grew. What we can say to-day is that the dentinal fibres running from the pulp tissue toward the enamel are formations of living matter, and that the whole basis-substance between the dentinal canaliculi is supplied with living matter too, in short is alive. The meshes of this reticulum contain a gelatinous, glue-yielding basis-substance, which is infiltrated with lime salts.

The dentine is a formation of connective tissue, and kindred with bone. In 1873 I was the first to assert that in the bone tissue not only the bone corpuscles but also the basis-substance is alive. I proved the same in regard to all varieties of connective tissue, which are composed of a continuous mass of living matter, pervading the whole body and not interrupted anywhere. It was an important view, I should say, for the consideration and study of the animal body as such. Up to that time the notion had prevailed that we are composed of cells, which were thought to be individuals, and that we are built up on the plan of a chimney or a dwelling, in which a number of bricks are set upon each other and stuck together with cement in order to build up the whole organization. With me there are no such cells, no such individual constituents of the body, but there is a continuity of living matter all through. One of the proofs of this view was found in the bone, and later on also in the dentine. This view being novel, and having the tendency to deal of opposition. You know that but very few men are capable of doing some thinking of their own. Most people, speaking in a general way, in any department of science, are scarcely doing more than chew the cud. What is accepted by the majority is considered as a dogma, and is adhered to with an obstinacy perfectly surprising. If a man appears who has no direct adherence to any school, and who tries to prove what he maintains in the laboratory under the microscope, comparatively few people can satisfy themselves of the correctness of his views, or the accuracy of the facts upon which his views are based. That was the case with me and my theories for a number of years; but an excellent histologist in Europe has now, without any hesitation, acknowledged their correctness, namely, Professor S. Stricker, of Vienna, formerly my teacher. Professor Stricker said it took him six years of work before he could satisfy himself of the truth of my assertions. Last year when in Vienna Prof. Stricker showed me some specimens of living tissues, particularly the cornea or anterior membrane which covers the eye, that had been brought to a slight degree of inflammation. If you look at such a specimen of a cornea, without further treatment, you can see the motion of the living matter all throughout the basis-substance. That is a strong proof of the correctness of my assertion that the basis-substance is alive also. The transparent animal tissues, especially the messentery of a freshly killed frog as shown by Prof. Spina, demonstrate the same thing.

It seems, therefore, that so long as we live we are changing the shape of our bodies all the time, either by growth or by locomotion that is going on throughout the tissues of the body. All tissues change their shape and place to a limited degree, and this view would enable us to understand the circulation of the nourishing material, its ups and downs within the teeth, in which we notice great variation in their chemical composition, as before mentioned, even in the space of a few weeks.

The next tissue which interests us is the enamel. Respecting the structure of the enamel, the notion prevailed that it was composed of prisms running longitudinally and obliquely with the axis of the tooth. It was maintained that these enamel prisms lie close against and in contact with each other, there being no interstices between them. Dr. Bodecker proved the existence of narrow interstices between the enamel prisms, and also that these interstices contained extremely minute fibres of living matter; about the same as those in the dentine, only on a very much smaller scale. The enamel fibrillæ which runs between the enamel prisms are extremely delicate to be sure, but they can be seen by the application of chloride of gold, and in temporary teeth even without the use of any re-agent. The younger the teeth the better you can see the course of the enamel fibrillæ. Dr. Bodecker has also shown that these delicate enamel fibrillæ send out off-shots toward the prisms and that the latter themselves are pervaded by a delicate reticulum, very much the same as the dentine. In all probability the enamel is traversed by living matter the same as the dentine. From the moment we become convinced that the enamel is a live tissue all the phenomena mentioned, the sensation which the seed of the raspberry or a delicate hair will produce, become explicable, we are at once in a position to understand that a tissue which is alive and traversed by living matter is endowed with properties of sensation. As we now know that the fibres that pervade the enamel and dentine are very probably in direct connection with the nerves present in the pulp, there is no difficulty in understanding the transmission of sensation from the periphery of the tooth to its centre.

The third tissue entering into the construction of a tooth is the cementum that covers the root. It is a structure similar to bone, and contains, especially in its lower portions, lacunæ filled with branching corpuscles, similar to bone-tissue and termed cement corpuscles; their off-shoots pervade the basis-substance, producing a continuous reticulum of living matter. What we know to-day to be cement corpuscles were, in previous years, considered as mere lacunæ, the hollow spaces of which send out off-shoots, the so-called canaliculi. The idea prevailed that they contained a liquid holding lime salts in solution. Others maintained that the contents were gaseous in nature. Neither of those theories is correct. Cementum is of the same structure as bone-tissue; its lacunæ contain living matter in the shape of protoplasm, the bone corpuscles, these being the centres from which a certain territory is supplied with living matter too.

What I want to prove, and have proved, is that the three hardest and densest tissues of the teeth, the cementum, the dentine and the enamel, are live tissues, so long as the tooth is in connection with the living organism; so long, at least, as the central part, or pulp, is present, in which we know there are a great many nerves and blood-vessels. If the pulp be destroyed, we have still a live connection with the surrounding pericementum, which is composed of bundles of fibrous tissue, surrounding the root of the tooth, and containing blood-vessels and nerves. We necessarily come to the conclusion that even an apparently dead tooth, that is, a tooth deprived of its pulp, must to a certain extent be supplied with nourishing material and be alive, at least in the peripheral portions of the cementum. How far that life goes nobody can say, for it is impossible to determine under the microscope the difference between living matter that is still alive and living matter that has become dead. The fibres are preserved, and so is the reticulum, and we may infer that there is a degree of vitality left in the cementum even after the destruction of the pulp.

I now come to the only soft tissue that enters into the construction of a tooth, the pulp tissue. This was also the subject of very careful researches in my laboratory by Dr. Bodecker. It was known that it consists of a delicate myxomatous tissue, which at the points of intersection shows thickenings called nuclei, evidently the centres of nutrition, or rather centres of life. There is a reticulum in the shape of delicate fibrillæ, between which lies the basis substance that we call myxomatous. Both the fibres and the myxomatous basis-substance are the seat of life, being pervaded by living matter. The pulp is one of the few myxomatous tissues in the human body that remain myxomatous in the adult. While in the embryo there is present a great deal of myxomatous tissue, nay, originally every variety of connective tissue is myxomatous in nature, about the time of birth we have only the umbilical cord, the placenta, the vitreous body of the eye, and the pulp tissue. The tissue of the pulp remains myxomatous during life. We know that as soon as the pulp assumes the features of fibrous connective tissue we must consider this condition as morbid. A pulp that has once been the seat of inflammation will become hyperplastic and cicatricial or fibrous. The myxomatous tissue of the pulp is pervaded by a large number of nerves. They run in bundles in the shape of medullated nerves, which, as they approach the periphery, become non-medullated and send numerous off-shoots toward the periphery of the pulp tissue, where the dentine is located. Here we find bodies in rows, having the aspect of epithelia, called odontoblasts, which are invariably present whenever the tooth is fully developed and is in a condition of comparative rest. I lay stress upon this fact, for it seems, as shown by an article that I received a few moments ago upon the development of the enamel, that it is very difficult for some histologists to understand that a tissue once formed is not permanent, and does not retain a permanent form during life-time. What I said about the tissues changing their shape and place during life in general, holds good with respect to the single elements. We have been told that after the so-called cell has formed it could not change farther. But, on the contrary, I am convinced that such changes occur through the life-time of the person, and invariably takes place during the growth of the tissues. I am convinced that these odontoblasts are formations to be found only at comparative rest, when there is no change going on toward a new formation or growth. As soon as such growth is going on, we see the odontoblasts no longer in the shape of epithelial bodies; they are transformed into medullary or embryonal corpuscles. We can prove that changes in the pulp tissue for the transformation of one tissue into another, namely, into dentine, can take place only through the intermediate stage of embryonal tissue and embryonal corpuscles.

Gentlemen, those of you who have not looked at the specimens in my laboratory probably will not have much faith in my assertions, especially in view of the fact that they are by no means universally accepted yet. I did my best last year in Europe to bring these views to the notice of the majority of the scientists in Berlin, Vienna and Heidelberg, but it was only in Vienna that they met a hearty approval, and this was owing to the fact that in Vienna resides the most advanced histologist of Europe, Professor Stricker. Neither in Berlin nor in Heidelberg were they advanced far enough to appreciate the value of these researches. In Heidelberg I spoke before an assembly of ophthalmologists and gave an exposition of the structure of the vitreous body which fills the posterior chamber of the eye, and the crystalline lens. I laid before them the proofs that the crystalline lens as well as the vitreous body, the former being epithelial and the latter myxomatous tissue, are alive. The prevailing idea there was that the vitreous body is a jelly-like mass, the same as in previous years. The idea prevailed that the umbilical cord was nothing but jelly, whereas I can demonstrate the presence of living matter through that jelly. I maintain that the vitreous body is a live tissue, and so is the crystalline lens. I assert that the lens undergoes changes, its epithelial structure, under certain conditions breaks down into medullary tissue and gives rise to new tissues, to connective tissue and even to bone. Two or three observers have agreed with me, but the great majority have not. I say that frankly because, although it is the aim of my life to show the correctness of these views, I am fully prepared to wait at least ten years yet for their acceptance. Professor Stricker, in a criticism on the German edition of my book, expresses his opinion that in the next ten years these, my views, will replace the old cell-doctrine. Perhaps it will take much longer. Perhaps I shall not be living when they are accepted. But one thing I can say, which is that so long as I live I propose to work and fight for their acceptation. When I returned from Europe last year, a friend said to me, "I traced your work through Europe; you have fought as hard as Sampson against the Philistines." I replied that the result was not fully satisfactory, perhaps because I did not work with the jaw-bone of an ass, as did old Sampson.

This little diversion from my subject was merely intended to demonstrate that transformations and transitions of tissues and elements are going on throughout life-time. Here you see (drawing on black-board) the odontoblasts at the periphery of the pulp tissue. It has been asserted by Tomes that the dentinal fibrillæ arise from the odontoblasts, being their direct off-shoots. This is correct so long as the odontoblasts resemble epithelia; but as soon as there is a transformation of them into medullary or embryonal corpuscles, there arises a number of delicate fibrillæ between the former odontoblasts. In many instances where the odontoblasts were fully developed, the fibrillæ were traced from their periphery, but when the odontoblasts have changed into medullary elements, we can see the fibrillæ which we call dentinal fibrillæ, run between the odontoblasts, and either directly or indirectly through the reticulum of living matter in connection with nerve fibres. It has been maintained that this transition is a direct one, but this seems not to be the case, at least with majority of the pulps I have seen. The question is of no importance, however, because both the fibrillæ and the nerves are fermentations of living matter. To-day it appears that is was no mistake of Tomes to maintain that the dentinal fibrillæ are nerves, for even to-day we know no boundary line or sharp distinction between the fibres of living matter, termed dentinal fibrillæ, and those termed nerves. There is a continuity of living matter from the periphery of the enamel of a tooth through the dentine with the nerves of the pulp, and, of course, with the brain and the sensual organs.

These are facts, at least acknowledged in my laboratory, and I hope by all of you who have attended my lectures. And now the question arises, what is all that good for? to be sure, the scientist does not care much about the usefulness of any discovery he makes. He takes the facts as he finds them, and very rarely thinks of the practical value that may arise from them. Nevertheless, I dare say that the practical value of the facts laid down before you is very great indeed. I believe every one of you will understand that it makes a great deal of difference in the practice of your profession, whether one takes it for granted that a tooth is merely a piece of chalk, an accumulation of lime salts, or whether one regards it as a live tissue. Even the enamel, which was formerly considered as being something like a very refined calcareous or crystalline material, we have proved to be a live tissue. It seems to me that every dentist must recognize the importance of this discovery. In former years to remove at random a certain amount of the enamel, to trim it off in order to increase the space between the teeth and thereby facilitate access to a carious cavity, was considered as but a trifling matter, and perfectly legitimate. To-day the cutting of the enamel must be restricted to a great extent. As soon as we know that the enamel is alive, and the best protective tissue of the teeth, we must become extremely careful in handling that tissue. We can no longer remove the enamel simply to increase the space between the teeth; we should rather remove a superfluous tooth and let nature repair the injury, than remove the enamel. Certainly we will not remove the enamel in order to gain easy access to a carious cavity unless it is absolutely necessary.

A second practical gain is the knowledge that every foreign body we introduce into the tissues of the teeth will have its future action and re-action upon them. Every filling that the dentist brings into a tooth is a foreign body, and as such necessarily excites more or less inflammation. A hundred years ago the celebrated German philosopher and poet, Goethe, made some examinations of the tusks of elephants, which were the seat of gun balls. We know that the target of the elephant hunter is the socket of the eye. That being missed the ball not infrequently enters the animal's tusk: and it was found that a gun ball lodged in an elephant's tusk, and remaining there for months or years, produces a remarkable change in the dentine. This is easily recognized by the naked eye of the manufacturer of billiard balls from these tusks. The dentine of a tusk in which a gun ball has been lodged presents a changed appearance; it has become brittle and unfit for to be turned into billiard balls because it has lost its elasticity. Here is a proof that a foreign body produces quite a marked change in the dentine. A filling that you introduce into the enamel or the dentine for a beneficial purpose will work exactly on the same plan; it is a foreign body, and, therefore, will excite reaction and an inflammatory process, the result of which will be a change of the tissue. This may, I admit, be beneficial in the highest degree. Suppose the inflammation around a filling leads to changes which render the dentine more firm and solid; that I would consider an excellent result of the filling. But suppose, on the contrary, that the reaction caused by the filling should be so intense as to excite a severe inflammation around it. The whole tissue being built up of living matter, the consequence would be that the inflammation would spread toward the pulp chamber, the pain, in many cases, becoming so great as to demand the removal of the filling, even though it be at a considerable distance from the pulp chamber. Evidently it is of importance to know how much living matter is present in any given case; in other words, how much of the material of the teeth is lime salts and how much live tissue. We know that the temporary teeth have very large canaliculi and very extenslve formations of living matter, with a small proportion of lime salts, in comparison with the teeth of adults. We must, therefore, take into consideration this state of things whenever we put a filling into a temporary tooth. Although I am not a dentist, I should say, speaking from a theoretical standpoint, that I would not consider it good practice to put a gold filling in a temporary tooth, the probability being that a solid gold filling will produce too great a re-action and inflammation will arise. In the teeth of adults you find a much greater degree of hardness and consistency, which is readily discovered when you work upon them with instruments; and the teeth of adults again differ in hardness. Therefore, from a theoretical standpoint, I should urge upon dentists to be cautious and not confine themselves to one filling material, such as gold alone, but to have on hand different materials adapted to the needs of different individuals and the requirements of different kinds of teeth.

It has been maintained that civilization and culture have a great deal to do with developing the process of caries in teeth. Such was the assertion of an excellent New York dentist, Dr. Kingsley. Now, if we say that the more culture advances the more prone are the teeth to become carious, I think we have not exhausted the question, and have not reached the bottom. We know that with advancing culture bodily strength usually decreases. We know that strong, iron constitutions are found in people who fight all their life-times against the influences of the weather and climate, people who work hard and do not over-feed themselves · whereas people who have highly cultivated their minds, and at the same time neglected to exercise their bodies as they ought to, frequently do show a noticeable decay from generation to generation. That is a point settled in the last century by J. J. Rousseau and other encyclopœdists. If among highly cultured people caries of the teeth is a prevailing feature, we must come to the conclusion that the organism is lacking in lime salts, and the process of caries thereby fostered. Caries has been proved to be an inflammatory process, which is assisted by low vegetable organisms, leptothrix and baccilli; and very probably such a morbid process is more easily and rapidly progressing in teeth poorly supplied with lime salts than those of strong and healthy people.

Lastly, the value of these new views will be apparent as soon as we study the history of the development of the teeth. According to the cell doctrine previously held, the tissues of the body being once formed cannot change; but with such views the development of the teeth was not explicable. To-day we are able to understand than an already formed tissue may change, return to its embryonal condition and give rise to new tissues; and we must realize that the growth of tissues can take place only through an intervening stage of medullary tissue.

I have laid down these novel principles in my microscopical morphology, and there for the first time brought them before the public. Both English and German editions of the book have been issued. I thought that publication would help to make the new views more popular; but after a time I came to the conclusion that outside of the laboratory, and direct demonstration of the facts, there is little hope of spreading the new ideas. Even comparatively good histologists outside of the laboratory, who are not pains-taking enough to investigate the matter, especially in the way in which I teach it by educating the eye to see and requiring the hand to draw everything upon paper that is seen, hardly ever will become convinced of the correctness of these views. A histologist in New York went so far as to decline the honor that America has in accepting this new doctine. My laboratory has been a great success. I have just finished the eightieth class, and that means at least 800 attendants in a little less than ten years. I think that proves at least one thing, namely, that American histologists are ahead of those of other nations, that they can see more and be taught easier. The gentleman I referred to thinks America needs no such distinction, and does not even consider the new doctrine a progressive step.

I bring these facts before you, gentlemen, in order to frankly show you the difficulty of teaching new truths. Every one who is anxious to learn anything about a novel doctrine have a teacher in the first place. I had my teacher, and I am the teacher of others who will, in their turn, be the teachers of others still. It is, therefore, with the view of asking those of you who are interested in these things, and desire to learn how to demonstrate them, to come to my laboratory. Quite a number of the gentlemen here present have been there, and I dare say that none of them have left it dissatisfied. I did not bring a microscope here, nor anything in the way of demonstrating these facts, for I know it is futile to attempt to do it at once. The eye must first be educated to a certain extent, and when you are prepared to see you can see what there is to be seen. One of the most important things in connection with dentistry is the use of the microscope. I believe that as the knowledge of these new views broadens the mind, and more attention is given to the study of microscopy, you shall look upon the teeth in an entirely different light from what you did before.—*Proceedings of New Jersey State Dental Society.*

